# Fingerprints of Multiple Electron Scatterings in Single-Layer Graphene

**DOI:** 10.1038/srep22570

**Published:** 2016-03-03

**Authors:** Minbok Jung, So-Dam Sohn, Jonghyun Park, Keun-U Lee, Hyung-Joon Shin

**Affiliations:** 1 School of Materials Science and Engineering, Ulsan National Institute of Science and Technology (UNIST), UNIST-gil 50, Ulsan 44919, Republic of Korea; 2 Center for Multidimensional Carbon Materials, Institute for Basic Science (IBS) Center for Multidimensional Carbon Materials, Institute for Basic Science (IBS), UNIST-gil 50, Ulsan 44919, Republic of Korea; 3 KIST-UNIST Ulsan Center for Convergent Materials, Ulsan National Institute of Science and Technology (UNIST), UNIST-gil 50, Ulsan 44919, Republic of Korea

## Abstract

The electrons in graphene exhibit unusual behaviours, which can be described by massless Dirac quasiparticles. Understanding electron scattering in graphene has been of significant importance for its future application in electronic devices because electron scattering determines electrical properties such as resistivity and electron transport. There are two types of electron scatterings in graphene: intervalley scattering and intravalley scattering. In single-layer graphene, to date, it has been difficult to observe intravalley scattering because of the suppression of backscattering resulting from the chiral nature of the electrons in graphene. Here, we report the multiple electron scattering behaviours in single-layer graphene on a metallic substrate. By applying one- and two-dimensional Fourier transforms to maps of the local density of states, we can distinguish individual scattering processes from complex interference patterns. These techniques enable us to provide direct evidence of intravalley scattering, revealing a linear dispersion relation with a Fermi velocity of ~7.4 × 10^5^ m/s.

Graphene, a two-dimensional (2D) honeycomb carbon lattice, has drawn considerable interest since its first successful isolation in a single layer^1^. Graphene has been proposed as a promising material for future electronic devices owing to its unique electrical properties^1^,^2^,^3^,^4^,^5^. When electrons are confined at graphene or noble metal surfaces, the incident and scattered electrons around imperfections produce standing wave patterns^6^,^7^,^8^,^9^,^10^,^11^. Electron scatterings in graphene are characterized by intervalley and intravalley processes, which occur on non-equivalent Dirac cones^8^. One of the central issues of electron scattering process in graphene is intravalley scattering in single-layer graphene (SLG). Quasiparticle interference associated with intravalley backscattering is known to be forbidden in SLG owing to the pseudospin and chirality of its electrons^8^,^9^,^12^. In contrast, bilayer graphene (BLG), which has a different distribution of pseudospin textures, allows intravalley scattering^8^. Experimentally, intra- and intervalley scatterings can be probed by Fourier-transform scanning tunnelling spectroscopy (FT-STS)^8^,^9^,^12^,^13^, which allows one to understand the complex electron scattering behaviours from quasiparticle interference patterns in various systems^14^,^15^,^16^,^17^. Despite intravalley scattering being forbidden in SLG, however, some theoretical studies predicted that the localized impurity potential in SLG could generate long-wavelength Friedel oscillations through intravalley scattering^18^,^19^,^20^,^21^. Experimental observations of intravalley scattering indicate that it is a rare but feasible process in SLG^11^,^22^. In this article, we report direct evidence of intravalley scattering on SLG/Cu(111). We utilize dipole moments developed at the atomic steps of the underlying Cu substrate to induce a localized potential in SLG for intravalley scattering. On a metallic substrate, the electronic states of both the graphene and substrate affect the electron scatterings at surface, resulting in intervalley, intravalley, and interband scatterings. We can visualize a linear dispersion relation and verify each scattering process by applying one- and two-dimensional Fourier transformations (FTs) to the scanning tunnelling spectroscopy (STS) map.

We investigated the electronic structure of SLG on Cu(111) by using scanning tunnelling microscopy (STM) and STS at cryogenic temperatures. Figure 1a shows an STM image of a SLG island on Cu(111). The graphene edge and defects function as scattering centres, resulting in the formation of interference patterns in STM topography^6^,^7^. The periodicity of modulation changes according to the sample bias because the standing wave pattern is determined by the energy of electrons, *i.e*. the Fermi wave vector of electrons. Figure 1b shows a one-dimensional (1D) STS map plotting the differential conductance d*I/*d*V* as a function of sample bias and position along the line marked in Fig. 1a. The d*I*/d*V*(*r*, *V*) map represents the spatial variation of the local density of states (LDOS). The horizontal and vertical modulations in the 1D-STS map correspond to the energy-resolved standing waves and LDOS at specific positions, respectively. In general, dispersion relation can be resolved by measuring the wave vector as a function of energy, and this method has been quite useful and widely used in previous studies for verifying dispersion relations^6^,^7^,^10^,^11^,^13^,^22^,^23^. However, it requires a large data set to fit the dispersion relation precisely.

In this study, instead of acquiring STS maps at various energies, we applied a 1D-FT to the d*I*/d*V*(*r*, *V*) map through the x-axis to obtain the dispersion relation. By applying a FT through each horizontal line in the d*I*/d*V*(*r*, *V*) map, we can illustrate a d*I*/d*V* map in the momentum space (Fig. 1c). The wave vector, ***k***, of standing waves is derived using *k* *=* *π/λ* because the imaged standing waves represent the charge density waves. Interestingly, the dispersion relation is parabolic: *E* = *E*_0_ + (*ћk*)^2^/2m^*^, where *E*_0,_ *ћ* and m^*^ are the binding energy of the surface state, Planck’s constant divided by 2π and the effective mass of an electron, respectively. Through the parabolic fitting, we obtain *E*_0_ and m^*^ as –0.30 ± 0.02 eV and (0.393 ± 0.004)*m*_e_, respectively. For reference, we obtained the dispersion relation of a clean Cu(111) surface using 1D FT-STS (Fig. 1d). The clean Cu(111) surface also shows a parabolic dispersion relation, in which *E*_0_ and m^*^ are –0.44 ± 0.02 eV and (0.396 ± 0.004)*m*_e_, respectively, in good agreement with previous measurements^6^,^24^,^25^,^26^. This means that the standing wave observed in SLG (Fig. 1a) is mainly attributed to the upward shift of the surface state of Cu(111), rather than to the electronic states of graphene. Graphene does not affect the effective mass of electrons in the underlying Cu(111) surface, implying weak electronic coupling between the graphene and Cu(111). As SLG is electronically transparent near Fermi level (*E*_F_)^23^, the surface state of the underlying Cu substrate appears in STS. In general, the energy level of the Dirac point (*E*_D_) has been assigned as a “dip” in d*I*/d*V*. As noted above, when graphene is prepared on a metal surface having surface states, it is inappropriate to determine *E*_D_ in such a manner. If the shifted surface state and *E*_D_ are located close to each other, it is difficult to distinguish the two by using normal d*I*/d*V* spectra.

Although there are additional complex features in the 1D-STS of graphene, we could not resolve the electronic structure of SLG. Neither the SLG edge nor the extrinsic impurities cause intravalley scattering in our results (Fig. S1). Graphene does not have specific electronic structures near the **Γ** point of ***k***-space at *E*_F_. Unlike the normal electron scattering processes on a noble metal surface, the intravalley scattering and tunnelling density of states (TDOS) are sensitive to the propagating direction of electron waves with respect to the graphene lattice^20^. Therefore, the orientation of line spectroscopy is expected to be an important factor for the observation of intravalley scattering in 1D FT-STS map of SLG (Fig. S2). In this regard, we examined the electron scattering around a step of the underlying Cu substrate along the specific direction. Figure 2a shows a d*I*/d*V*(*x, y*) map of SLG lying flat on the Cu step. From the 2D-FT of the d*I*/d*V*(*x, y*) map, we can verify the contribution of wave vectors, which determine the observed interference patterns combining multiple scatterings (Fig. 2b). The asymmetric concentric rings at the corners of the first Brillouin zone in ***q***-space constitute evidence of intervalley scattering. In the case of intravalley scattering, electron scattering in a single Dirac cone should be mirrored on the **Γ** point of ***q***-space in the 2D FT-STS map^8^,^9^,^12^, hence a ring-type contour is expected at the centre. However, the shifted surface state of the underlying Cu(111) near *E*_D_ and other scattering processes make it difficult to distinguish intravalley scattering. We performed 1D line spectroscopy along the line marked in Fig. 2a, which is parallel to the line crossing the **Γ** and **K** points (Fig. 2b). By applying 1D-FT, we can successfully produce an *E-k* map revealing the parabolic dispersion relation of the Cu(111) surface state as well as the linear dispersion relation of the SLG (Fig. 2d). The dispersion relation (dotted line in Fig. 2d) originating from intravalley scattering is fitted by *E*(*k*) = ±*ħv*_F_*k* + *E*_D_, where *v*_*F*_ is the Fermi velocity. The *E*_D_ is located –300 meV below *E*_F_. It should be noted that *v*_F_ is twice the slope of a linear fit in 1D FT-STS because it reflects the diameter of constant-energy circles. Here, *v*_F_ is estimated as (7.36 ± 0.12) × 10^5^ m/s. As the wave vector of intervalley scattering is much larger than that of intravalley scattering, energy is found to be linearly dependent on the wave vector on the basis of the plot of the radius of the scattering ring contour at the **K** points in ***q***-space as a function of energy. The *v*_F_ estimated from the intervalley scattering has a similar value of (7.79 ± 0.93) × 10^5^ m/s (Fig. S3). There are two main reasons for the observation of the forbidden intravalley scattering in SLG. First, dipole moments develop at the step edge of Cu(111), which induce a localized electric field in graphene^27^. Second, when SLG grows on the Cu surface, it conformally covers Cu steps owing to van der Waals interaction and its high flexibility. Therefore, the corrugation of SLG at the Cu step can break the symmetry of the carbon lattice. Consequently, intravalley scattering becomes available, as predicted in a previous report^21^.

There is an additional feature in the 2D FT-STS map of SLG. Double ring contours appear at the **Γ** and **K** points in the 2D FT-STS (Fig. 2b). The double ring contours at the **K** points are caused by interband scattering between the Cu substrate and SLG (Fig. S3). We cannot observe double ring contours at the **Γ** point when the impurity density is low. Figure 3a shows an STM topography (left) and corresponding d*I*/d*V* map (right) of SLG/Cu(111). Here, many defects also appear in STM image. From the atomically resolved STM image (inset of Fig. 3a), we can confirm that the point defects are impurities on the Cu(111) surface rather than those in SLG. Indeed, we can also observe two concentric rings at the **Γ** point (Fig. 3b). The outer ring originates from the surface state of Cu(111), whereas the inner ring is attributed to electron scattering caused by the impurities in bulk Cu^28^,^29^,^30^. An additional ring results from the scattering of bulk state electrons with the ***k*** vector around the ‘neck’ of the bulk Fermi surface. The inverse-FT image clearly visualizes these contributions (Fig. 3c). For the surface-state scattering, standing waves show a depression at the impurity positions. In contrast, the bulk-state scattering exhibits the maximum intensity at these locations, and the interference patterns are localized near impurities. The dispersion relation associated with the bulk-state scattering shows anomalous behaviour (Fig. 3d), as it diverges above the *E*_F_. Below the *E*_F_, electrons from the surface state of Cu tunnels into the STM tip, leaving holes at the bottom of the surface state^31^. In this process, electrons with higher energy at the surface state and the bulk state can pass through the hole by inter- and intraband transitions which enhance decay rate of electrons^31^,^32^. The radius of the ring reflects the surface-parallel component of the wave vector, ***k***_***‖***_. Above the *E*_F_, the electron decay rate becomes comparable to the tunnelling rate, and the contour becomes broader and blurred at a constant radius. Furthermore, the surface-normal component ***k***_**⊥**_ increases, while ***k***_***‖***_ is confined to the projected bulk states, *i.e*. the radius of neck of Fermi surface. From the 1D FT-STS, we can also confirm the dispersion relation for the bulk-state scattering, the vertex of which is located at ~–0.55 eV (Fig. 1c).

Our results demonstrate that the apparent quantum interference pattern is generated from the different origins of multiple scattering processes in weakly interacting SLG/metal systems. The electronic states of both the graphene and substrate are preserved well even after contact, revealing simultaneously linear and parabolic dispersion relations. Imperfections in the underlying substrate, however, have a considerable effect on the electron scattering in the SLG: the imperfections are sufficiently strong to allow intravalley scattering in the SLG. We believe that our results can further the understanding of multiple electron scattering processes in SLG and provide a fundamental basis for understanding other hetero-junction systems of different electronic structures, such as topological insulators, molecular films, and transition metal dichalcogenides (TMDs).

## Methods

All experiments were performed using an ultrahigh-vacuum low-temperature scanning tunnelling microscope (SPECS, JT-STM) at 1.2 K. The base pressure of our system is less than 4.5 × 10^−11^ Torr. A Cu(111) single crystal was cleaned by several cycles of sputtering and annealing. After cleaning the substrate, SLG was grown through an Ar-assisted growth method^33^,^34^. Ethylene (99.8%, Sigma-Aldrich) and Ar gases were introduced into the preparation chamber at a pressure of 1.5 mTorr. The partial pressure of ethylene was 1.5 × 10^−4^ Torr. The temperature of the substrate was kept at 1073 K for 10 min. We measured the differential conductance, d*I*/d*V* with a lock-in detection technique by applying 5 ~ 50 mV (r.m.s.) modulation to the sample bias voltage at 727 Hz. In the 1D FT-STS maps, we removed the data at *k* = 0 for a clear visualization.

## Additional Information

**How to cite this article**: Jung, M. *et al*. Fingerprints of Multiple Electron Scatterings in Single-Layer Graphene. *Sci. Rep*. **6**, 22570; doi: 10.1038/srep22570 (2016).

## Supplementary Material

Supplementary Information

## Figures and Tables

**Figure 1:**
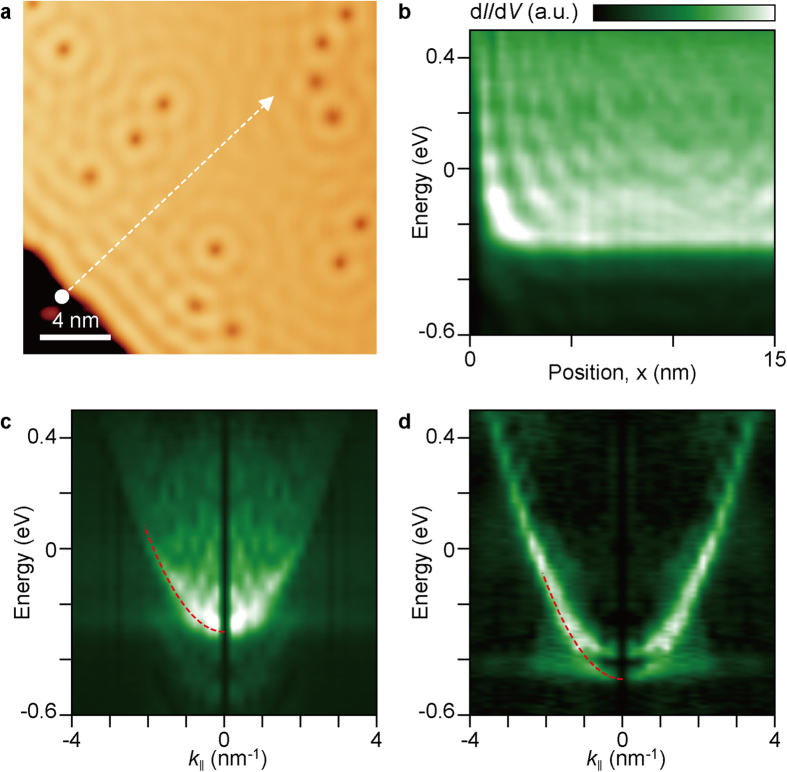
Multiple electron scatterings at the edge of SLG on Cu(111). (**a**) Topographic STM image of SLG on Cu(111) (*V*_sample_ = 0.1 V, sample bias; *I*_tunnel_ = 0.5 nA, tunnelling current). (**b**) 1D-STS map of SLG along the dashed line in **a**. (*V*_sample_ = 0.05 V; *I*_tunnel_ = 2.0 nA) (**c**) Fourier-transformed map of **b** as a function of *k* and the sample bias voltage. (**d**) 1D FT-STS map of a clean Cu(111) surface. The dashed parabolic curves marked in **c** and **d** indicate the dispersion relations of Cu surface states.

**Figure 2:**
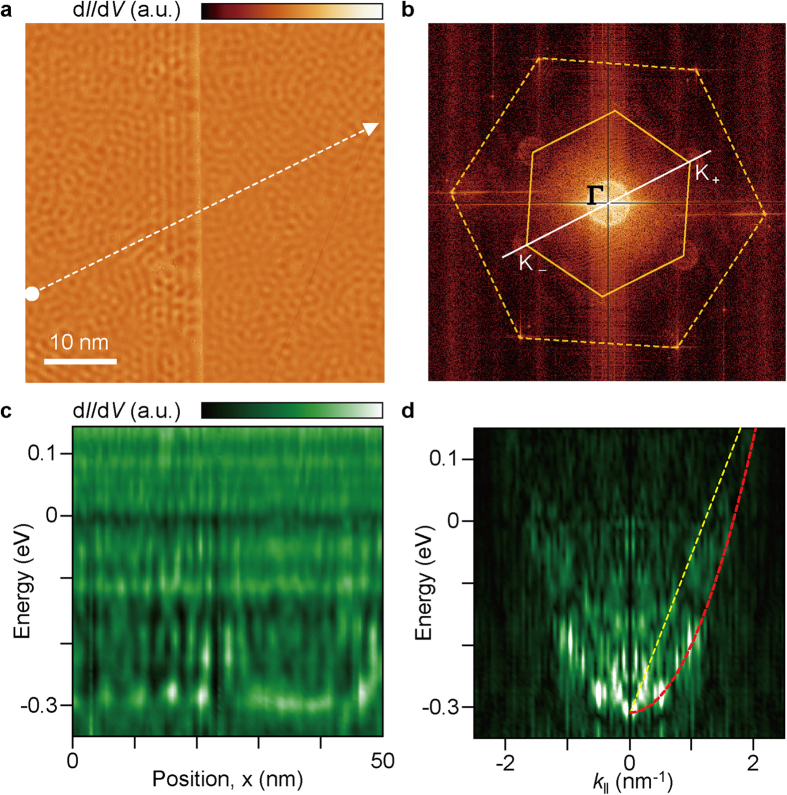
Intravalley scattering in SLG at a step of the underlying Cu substrate. (**a**) Spectroscopic map of d*I*/d*V* at 0.1 V for SLG lying across a step of the Cu substrate. The dashed line in **a** is parallel to the dashed line in (**b**), which passes through the **Γ** and **K** points. (**b**) ***q***-space map of interference patterns obtained from the 2D-STS map of (**a**) Inner and outer hexagons represent the first Brillouin zone and the reciprocal lattice of graphene, respectively. (**c**) 1D-STS map along the dashed line marked in (**a**) (*V*_sample_ = 0.3 V; *I*_tunnel_ = 1.0 nA). (**d**) 1D FT-STS map of **b** showing the linear dispersion relation of SLG (yellow dashed line) and parabolic dispersion relation of Cu(111) (red dashed curve).

**Figure 3:**
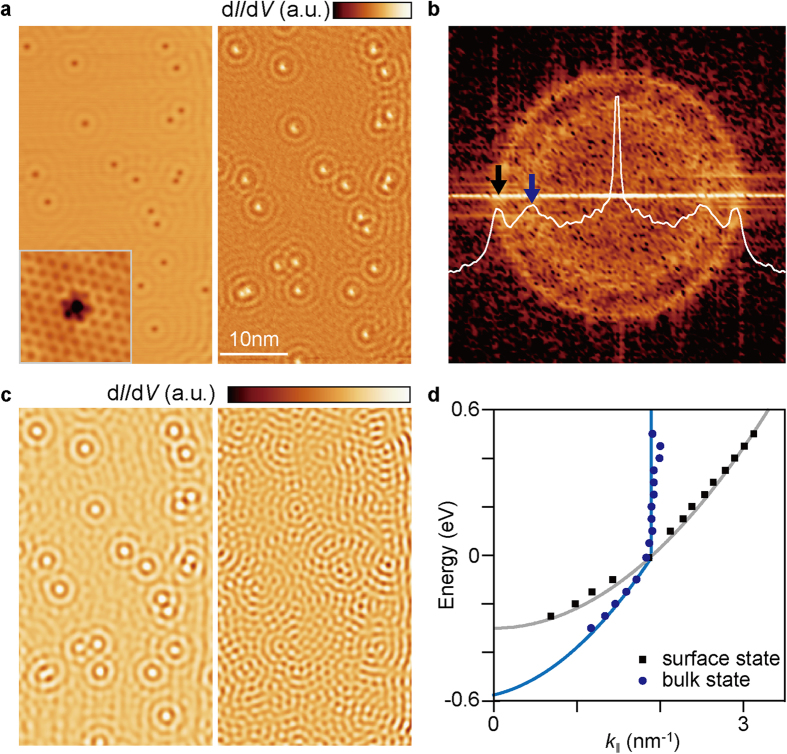
Bulk-state electron scattering at Cu substrate below SLG. (**a**) Topographic STM image (left) and corresponding d*I*/d*V* map (right) of SLG (*V*_sample_ = 0.3 V; *I*_tunnel_ = 2.0 nA). The inset in (**a**) shows a high-resolution STM image of SLG with an intrinsic impurity in the underlying substrate (2 × 2 nm^2^; *V*_sample_ = 0.05 V; *I*_tunnel_ = 2.0 nA). (**b**) Double-ring-type interference patterns at the **Γ** point of ***q***-space, acquired from the 2D-FT of the d*I*/d*V* map in **a** (7 × 7 nm^−2^). Plot shown in **b** is an angular average of the interference patterns at the **Γ** point. Black and blue arrows indicate the outer (surface-state scattering) and inner (bulk-state scattering) rings. (**c**) Inverse-Fourier-transformed interference patterns of bulk-state (left) and surface-state (right) scatterings. (**d**) Energy dispersions for bulk-state (blue) and surface-state (black) scatterings as functions of *k*, as determined from the 2D FT-STS maps at various energies.
