# Supplementary material for: Fingerprints of Multiple Electron Scatterings in Single-Layer Graphene
Source: Sci Rep. 2016 Mar 3;6:22570. doi: 10.1038/srep22570 (PMC4776258; doi:10.1038/srep22570)
Supplement: Supplementary Information [file srep22570-s1.pdf]

Supplementary Information for

Fingerprints of Multiple Electron Scatterings in Single-Layer Graphene

Minbok Jung[1,2], So-Dam Sohn[1], Jonghyun Park[1], Keun-U Lee[1,3], Hyung-Joon Shin[1,2,3*]

[1]School of Materials Science and Engineering, Ulsan National Institute of Science and Technology (UNIST), UNIST-gil 50, Ulsan 44919, Republic of Korea

[2]Center for Multidimensional Carbon Materials, Institute of Basic Science (IBS), UNIST-gil 50, Ulsan 44919, Republic of Korea

[3]KIST-UNIST Ulsan Center for Convergent Materials, Ulsan National Institute of Science and Technology (UNIST), UNIST-gil 50, Ulsan 44919, Republic of Korea

[*]To whom correspondence should be addressed. Email: shinhj@unist.ac.kr

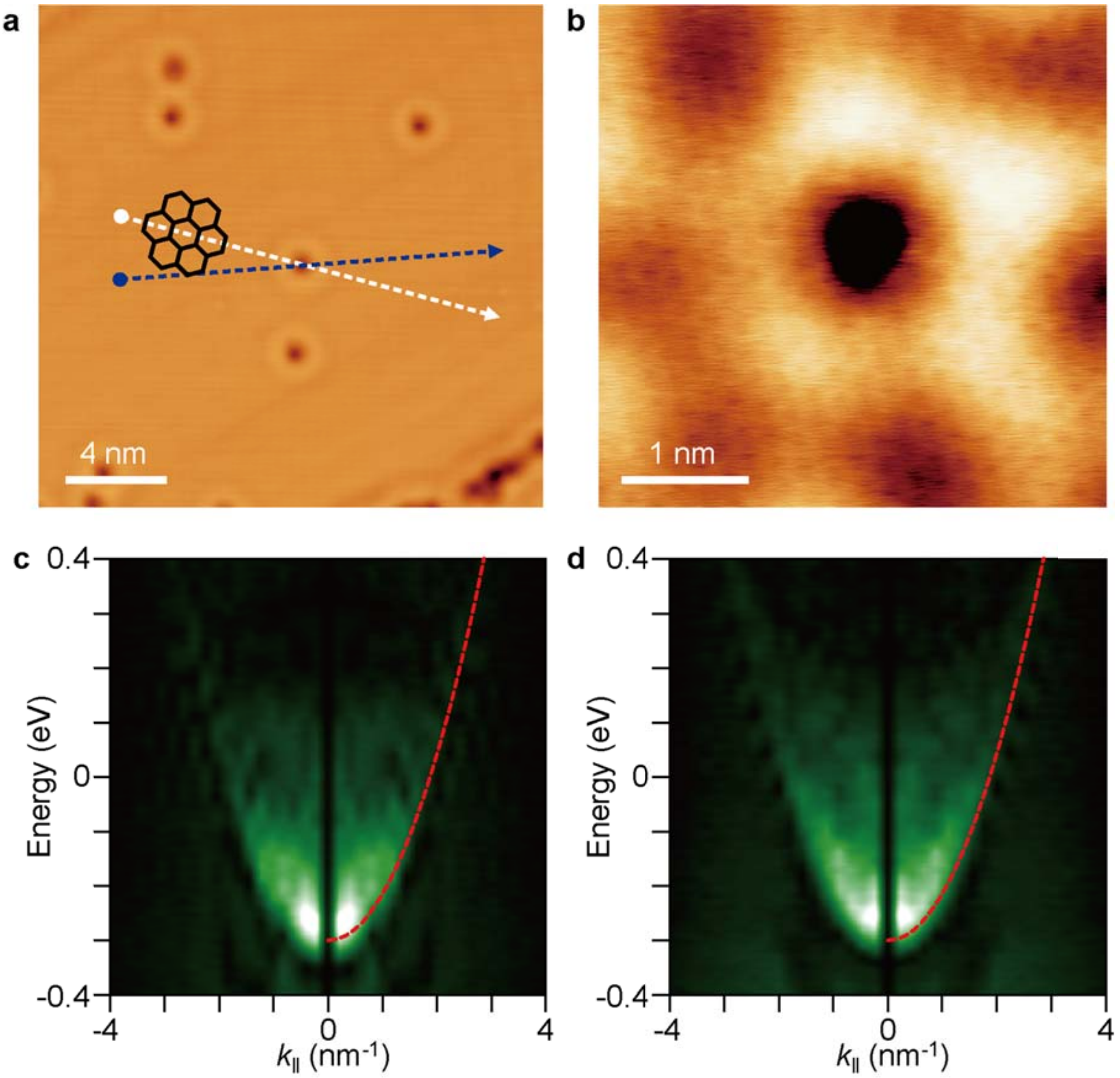

Figure S1. Multiple electron scatterings in SLG at a point defect of the underlying Cu substrate. (**a**) Topographic STM image of a SLG on Cu(111) (V_{sample} = 0.5 V; I_{tunnel} = 1.0 nA). The hexagons indicate the orientation of graphene lattice (V_{sample} = 0.005 V; I_{tunnel} = 5.0 nA). (**b**) High-resolution STM image showing the honeycomb structure of graphene. (**c**), (**d**) 1D FT-STS maps obtained along the white (**c**) and the blue (**d**) lines in **a**. The dashed parabolic curves marked in **b** and **c** indicate the dispersion relations of Cu(111).

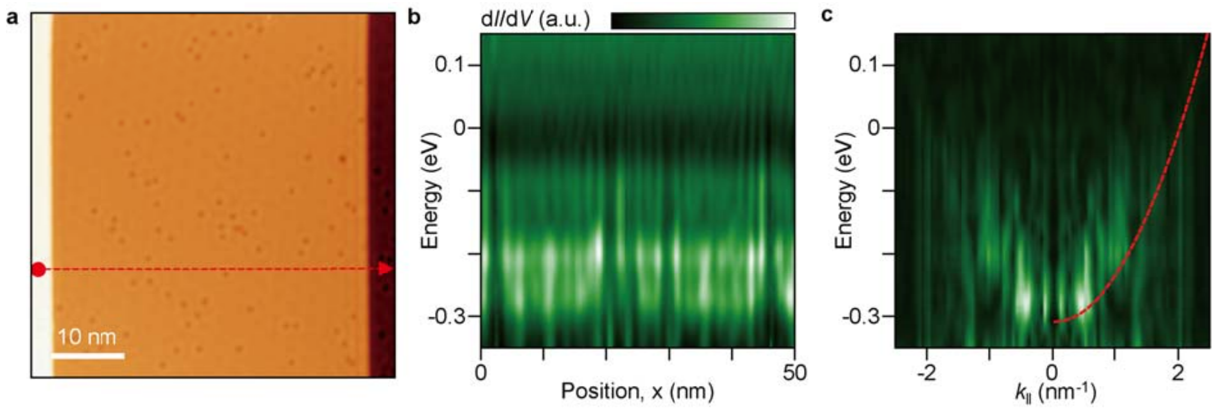

Figure S2. Orientation-dependent 1D FT-STS map of SLG. (**a**) Topographic STM image of a different region of the SLG in Fig. 1**a** (V_{sample} = 0.2 V; I_{tunnel} = 0.5 nA). The dashed line is tilted about 23° from the $\overline{\mathbf{\Gamma K}}$ direction. (**b**) 1D-STS map along the dashed line marked in **a**. (**c**) 1D FT-STS map of **b**. The dashed curve represents the parabolic dispersion relation of Cu(111).

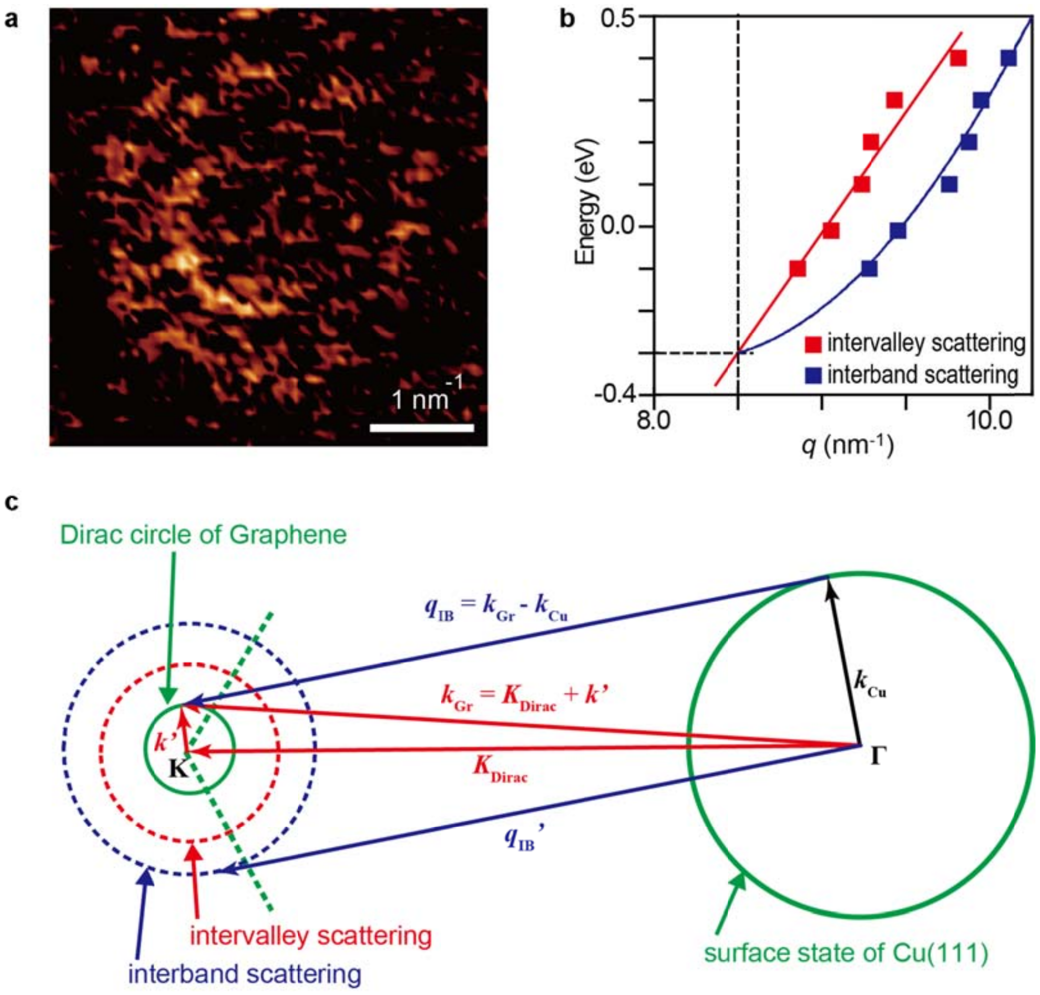

Figure S3. Intervalley and interband scatterings at SLG on Cu. (**a**) Double-ring-type interference patterns at the **K** point of ***q***-space, acquired from the 2D-FT of the d*I*/d*V* map in Fig. 3**a**. (**b**) Energy dispersions for intervalley (red) and interband (blue) scatterings as a function of *k*, as determined from the 2D FT-STS maps at various energies. (**c**) Schematic representation of the interband scattering process between SLG and Cu. Green circles indicate constant-energy rings of the system in ***k***-space, and $\boldsymbol{k}_{\mathbf{Gr}}$ and $\boldsymbol{k}_{\mathbf{Cu}}$ indicate wave vectors in the electronic states of SLG and Cu(111), respectively. $\boldsymbol{K}_{\mathbf{Dirac}}$ and ***k***' are the crystal momentum and radius of the constant-energy circle in the Dirac cone, respectively, and $\boldsymbol{q}_{\mathbf{IB}}$ represents the wave vector of interband scattering between $\boldsymbol{k}_{\mathbf{Gr}}$ and $\boldsymbol{k}_{\mathbf{Cu}}$. Dashed blue circle indicates the interband scattering feature in ***q***-space, formed by $\boldsymbol{q}_{\mathbf{IB}}$**'** ($\boldsymbol{q}_{\mathbf{IB}}$ vector translated to the **Γ** point). Dashed red circle shows the intervalley scattering feature.
